# Functional normalization of 450k methylation array data improves replication in large cancer studies

**DOI:** 10.1186/s13059-014-0503-2

**Published:** 2014-12-03

**Authors:** Jean-Philippe Fortin, Aurélie Labbe, Mathieu Lemire, Brent W Zanke, Thomas J Hudson, Elana J Fertig, Celia MT Greenwood, Kasper D Hansen

**Affiliations:** Department of Biostatistics, Johns Hopkins Bloomberg School of Public Health, 615 N. Wolfe St, E3527, Baltimore, 21205 USA; Department of Epidemiology, Biostatistics and Occupational Health, McGill University, 1020 Pine Ave. West, H3A 1A2, Montreal, Canada; Douglas Mental Health University Institute, McGill University, 8875 Boulevard Lasalle, H4H 1R3, Verdun, Canada; Department of Psychiatry, McGill University, 1033 Pine Avenue West, H3A 1A1, Montreal, Canada; Ontario Institute for Cancer Research, 661 University Avenue, Suite 510, M5G 0A3, Toronto, Canada; Clinical Epidemiology Program, Ottawa Hospital Research Institute, 725 Parkdale Ave., K1Y 4E9, Ottawa, Canada; Departments of Molecular Genetics and Medical Biophysics, University of Toronto, 101 College Street, Rm 15-701, M5G 1L7, Toronto, Canada; epartment of Oncology, Sidney Kimmel Comprehensive Cancer Center, Johns Hopkins University, 550 N. Broadway, Baltimore, 21205 USA; Lady Davis Institute for Medical Research, Jewish General Hospital Montreal, 3755 Cote Ste-Catherine Road, H3T 1E2, Montreal, Canada; Department of Oncology, McGill University, 546 Pine Ave. West, H2W 1S6, Montreal, Canada; McKusick-Nathans Institute of Genetic Medicine, Johns Hopkins School of Medicine, 1800 Orleans St., Baltimore, 21287 USA

## Abstract

We propose an extension to quantile normalization that removes unwanted technical variation using control probes. We adapt our algorithm, functional normalization, to the Illumina 450k methylation array and address the open problem of normalizing methylation data with global epigenetic changes, such as human cancers. Using data sets from The Cancer Genome Atlas and a large case–control study, we show that our algorithm outperforms all existing normalization methods with respect to replication of results between experiments, and yields robust results even in the presence of batch effects. Functional normalization can be applied to any microarray platform, provided suitable control probes are available.

**Electronic supplementary material:**

The online version of this article (doi:10.1186/s13059-014-0503-2) contains supplementary material, which is available to authorized users.

## Background

In humans, DNA methylation is an important epigenetic mark occurring at CpG dinucleotides, which is implicated in gene silencing. In 2011, Illumina released the HumanMethylation450 bead array [1], also known as the 450k array. This array has enabled population-level studies of DNA methylation by providing a cheap, high-throughput and comprehensive assay for DNA methylation. Applications of this array to population-level data include epigenome-wide association studies (EWAS) [2,3] and large-scale cancer studies, such as the ones available through The Cancer Genome Atlas (TCGA). Today, around 9,000 samples are available from the Gene Expression Omnibus of the National Center for Biotechnology Information, and around 8,000 samples from TCGA have been profiled on either the 450k array, the 27k array or both.

Studies of DNA methylation in cancer pose a challenging problem for array normalization. It is widely accepted that most cancers show massive changes in their methylome compared to normal samples from the same tissue of origin, making the marginal distribution of methylation across the genome different between cancer and normal samples [4-8]; see Additional file 1: Figure S1 for an example of such a global shift. We refer to this as global hypomethylation. The global hypomethylation commonly observed in human cancers was recently shown to be organized into large, well-defined domains [9,10]. It is worth noting that there are other situations where global methylation differences can be expected, such as between cell types and tissues.

Several methods have been proposed for normalization of the 450k array, including quantile normalization [11,12], subset-quantile within array normalization (SWAN) [13], the beta-mixture quantile method (BMIQ) [14], dasen [15] and noob [16]. A recent review examined the performance of many normalization methods in a setting with global methylation differences and concluded: ‘There is to date no between-array normalization method suited to 450K data that can bring enough benefit to counterbalance the strong impairment of data quality they can cause on some data sets’ [17]. The authors note that not using normalization is better than using the methods they evaluated, highlighting the importance of benchmarking any method against raw data.

The difficulties in normalizing DNA methylation data across cancer and normal samples simultaneously have been recognized for a while. In earlier work on the CHARM platform [18], Aryee *et al.* [19] proposed a variant of subset quantile normalization [20] as a solution. For CHARM, input DNA is compared to DNA processed by a methylation-dependent restriction enzyme. Aryee *et al.* [19] used subset quantile normalization to normalize the input channels from different arrays to each other. The 450k assay does not involve an input channel; it is based on bisulfite conversion. While not directly applicable to the 450k array design, the work on the CHARM platform is an example of an approach to normalizing DNA methylation data across cancer and normal samples.

Any high-throughput assay suffers from unwanted variation [21]. This is best addressed by experimental design [21]. In the gene expression literature, correction for this unwanted variation was first addressed by the development of unsupervised normalization methods, such as robust multi-array average (RMA) [22] and variance-stabilizing normalization (VSN) [23]. As Mecham *et al.* [24], we use the term ‘unsupervised’ to indicate that the methods are unaware of the experimental design: all samples are treated equally. These methods lead to a substantial increase in signal-to-noise. As experiments with larger sample sizes were performed, it was discovered that substantial unwanted variation remained in many experiments despite the application of an unsupervised normalization method. This unwanted variation is often – but not exclusively – found to be associated with processing date or batch, and is therefore referred to as a batch effect. This led to the development of a series of supervised normalization tools, such as surrogate variable analysis (SVA) [25,26], ComBat [27], supervised normalization of microarrays (SNM) [24] and remove unwanted variation (RUV) [28], which are also known as batch effect removal tools. The supervised nature of these tools allows them to remove unwanted variation aggressively while keeping variation associated with the covariate of interest (such as case/control status). Unsurprisingly, batch effects have been observed in studies using the 450K array [29].

As an example of unwanted variation that is biological in origin, we draw attention to the issue of cell-type heterogeneity, which has seen a lot of attention in the literature on DNA methylation [30-34]. This issue arises when primary samples are profiled; primary samples are usually a complicated mixture of cell types. This mixture can substantially increase the unwanted variation in the data and can even confound the analysis if the cell-type distribution depends on a phenotype of interest. It has been shown that SVA can help mitigate the effect of cell-type heterogeneity [33], but other approaches are also useful [30-32].

In this work, we propose an unsupervised method that we call functional normalization, which uses control probes to act as surrogates for unwanted variation. We apply this method to the analysis of 450k array data, and show that functional normalization outperforms all existing normalization methods in the analysis of data sets with global methylation differences, including studies of human cancer. We also show that functional normalization outperforms the batch removal tools SVA [25,26], ComBat [27] and RUV [28] in this setting. Our evaluation metrics focus on assessing the degree of replication between large-scale studies, arguably the most important biologically relevant end point for such studies. Our method is available as the ‘preprocessFunnorm’ function in the minfi package [12] through the Bioconductor project [35].

## Results and discussion

### Control probes may act as surrogates for batch effects

The 450k array contains 848 control probes. These probes can roughly be divided into negative control probes (613), probes intended for between array normalization (186) and the remainder (49), which are designed for quality control, including assessing the bisulfite conversion rate (see Materials and methods and Additional file 1: Supplementary Materials). Importantly for our proposed method, none of these probes are designed to measure a biological signal.

**Figure 1:**
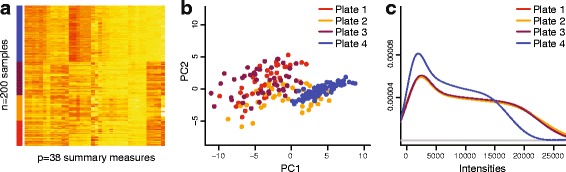
**Control probes acts as surrogates for batch effects.** **(a)** Heat map of a summary (see Materials and methods) of the control probes, with samples on the *y*-axis and control summaries on the *x*-axis. Samples were processed on a number of different plates indicated by the color label. Only columns have been clustered. **(b)** The first two principal components of the matrix depicted in **(a)**. Samples partially cluster according to batch, with some batches showing tight clusters and other being more diffuse. **(c)** The distribution of methylated intensities averaged by plate. These three panels suggest that the control probe summaries partially measure batch effects. PC, principal component.

Figure 1a shows a heat map of a simple summary (see Materials and methods) of these control probes, for 200 samples assayed on four plates (Ontario data set). Columns are the control measure summaries and rows are samples. The samples have been processed on different plates, and we observe a clustering pattern correlated with plate. Figure 1b shows the first two principal components of the same summary data and there is evidence of clustering according to plate. Figure 1c shows how the marginal distributions of the methylated channel vary across plates. This suggests that the summarized control probes can be used as surrogates for unwanted variation. This is not a new observation; the use of control probes in normalization has a long history in microarray analysis.

### Functional normalization

We propose functional normalization (see Materials and methods), a method that extends quantile normalization. Quantile normalization forces the empirical marginal distributions of the samples to be the same, which removes all variation in this statistic. In contrast, functional normalization only removes variation explained by a set of covariates, and is intended to be used when covariates associated with technical variation are available and are independent of biological variation. We adapted functional normalization to data from the 450k array (see Materials and methods), using our observation that the control probe summary measures are associated with technical variability and batch effects. As covariates, we recommend using the first *m*=2 principal components of the control summary matrix, a choice with which we have obtained consistently good results; this is discussed in greater depth below. We have also examined the contributions of the different control summary measures in several different data sets, and we have noted that the control probe summaries given the most weight varied across different data sets. We have found (see below) that we can improve functional normalization slightly by applying it to data that have already been background corrected using the noob method [16].

Functional normalization, like most normalization methods, does not require the analyst to provide information about the experimental design. In contrast, supervised normalization methods, such as SVA [25,26], ComBat [27], SNM [24] and RUV [28], require the user to provide either batch parameters or an outcome of interest. Like functional normalization, RUV also utilizes control probes as surrogates for batch effects, but builds the removal of batch effects into a linear model that returns test statistics for association between probes and phenotype. This limits the use of RUV to a specific statistical model. Methods such as clustering, bumphunting [12,36] and other regional approaches [37] for identifying differentially methylated regions (DMRs) cannot readily be applied.

### Functional normalization improves the replication between experiments, even when a batch effect is present

As a first demonstration of the performance of our algorithm, we compare lymphocyte samples from the Ontario data set to Epstein–Barr virus (EBV)-transformed lymphocyte samples from the same collection (see Materials and methods). We have recently studied this transformation [38] and have shown that the EBV transformation induces large blocks of hypomethylation encompassing more than half the genome, like what is observed between most cancers and normal tissues. This introduces a global shift in methylation, as shown by the marginal densities in Additional file 1: Figure S1.

We divided the data set into discovery and validation cohorts (see Materials and methods), with 50 EBV-transformed lymphocytes and 50 normal lymphocytes in each cohort. As illustrated in Additional file 1: Figure S2a, we attempted to introduce *in silico* unwanted variation confounding the EBV transformation status in the validation cohort (see Materials and methods), to evaluate the performance of normalization methods in the presence of known confounding unwanted variation. This has been previously done by others in the context of genomic prediction [39]. We normalized the discovery cohort, identified the top *k* differentially methylated positions (DMPs) and asked: ‘How many of these *k* DMPs can be replicated in the validation cohort?’ We normalized the validation cohort separately from the discovery cohort to mimic a replication attempt in a separate experiment. We identified DMPs in the validation cohort using the same method and the result is quantified using a receiver operating characteristic (ROC) curve where the analysis result for the discovery cohort is taken as the gold standard.

**Figure 2:**
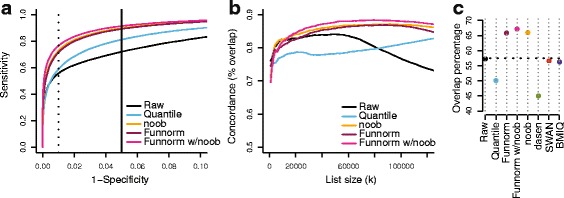
**Improvements in replication for the EBV data set.** **(a)** ROC curves for replication between a discovery and a validation data set. The validation data set was constructed to show *in silico* batch effects. The dotted and solid lines represent, respectively, the commonly used false discovery rate cutoffs of 0.01 and 0.05. **(b)** Concordance curves showing the percentage overlap between the top *k* DMPs in the discovery and validation cohorts. Additional normalization methods are assessed in Additional file 1: Figure S3. Functional normalization shows a high degree of concordance between data sets. **(c)** The percentage of the top 100,000 DMPs that are replicated between the discovery and validation cohorts and also inside a differentially methylated block or region from Hansen *et al.* [38]. DMP, differentially methylation position; EBV, Epstein–Barr virus; Funnorm, functional normalization; ROC, receiver operating characteristic.

To enable the comparison between normalization methods, we fix the number of DMPs across all methods. Because we know from previous work [38] (described as WGBS EBV data in Materials and methods) that the EBV transformation induces large blocks of hypomethylation covering more than half of the genome, we expected to find a large number of DMPs, and we set *k*=100000. The resulting ROC curves are shown in Figure 2a. In this figure we show, for clarity, what we have found to be the most interesting alternatives to functional normalization in this setting: raw data, quantile normalization as suggested by Touleimat *et al.* [11] and implemented in minfi [12] and the noob background correction [16]. Additional file 1: Figure S3a,b contains results for additional normalization methods: BMIQ [14], SWAN [13] and dasen [15]. Note that each normalization method will result in its own set of gold-standard DMPs and these ROC curves therefore measure the internal consistency of each normalization method. We note that functional normalization (with noob background correction) outperforms raw data and quantile and noob normalizations when the specificity is above 90% (which is the relevant range for practical use).

We also measured the agreement between the top *k* DMPs from the discovery cohort with the top *k* DMPs from the validation cohort by looking at the overlap percentage. The resulting concordance curves are shown in Figure 2b, and those for additional methods in Additional file 1: Figure S3c. The figures show that functional normalization outperforms the other methods.

We can assess the quality of the DMPs replicated between the discovery and validation cohorts by comparing them to the previously identify methylation blocks and DMRs [38]. In Figure 2c, we present the percentage of the initial *k*=100000 DMPs that are both replicated and present among the latter blocks and regions. We note that these previously reported methylation blocks represent large-scale, regional changes in DNA methylation and not regions where every single CpG is differentially methylated. Nevertheless, such regions are enriched for DMPs. This comparison shows that functional normalization achieves a greater overlap with this external data set, with an overlap of 67% compared to 57% for raw data, while other methods, other than noob, perform worse than the raw data.

### Replication between experiments in a cancer study

We applied the same discovery–validation scheme to measure performance as we used for the analysis of the Ontario-EBV study, on kidney clear-cell carcinoma samples (KIRC) from TCGA. In total, TCGA has profiled 300 KIRC cancer and 160 normal samples on the 450K platform. Therefore, we defined a discovery cohort containing 65 cancer and 65 normal samples and a validation cohort of 157 cancer and 95 normal samples (see Materials and methods).

**Figure 3:**
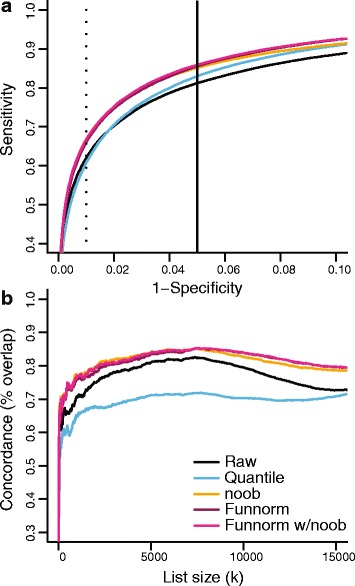
**Improvements in replication for the TCGA-KIRC data set.** **(a)** ROC curves for replication between a discovery and a validation data set. The validation data set was constructed to show *in silico* batch effects. **(b)** Concordance plots between an additional cohort assayed on the 27k array and the validation data set. Additional normalization methods are assessed in Additional file 1: Figure S4. Functional normalization shows a high degree of concordance between data sets. Funnorm, functional normalization; KIRC, kidney clear-cell carcinoma; ROC, receiver operating characteristic; TCGA, The Cancer Genome Atlas.

Our *in silico* attempt at introducing unwanted variation associated with batch for this experiment succeeded in producing a validation cohort where the cancer samples have greater variation in background noise (Additional file 1: Figure S1b). This difference in variation is a less severe effect compared to the difference in mean background noise we achieved for the Ontario-EBV data set (Additional file 1: Figure S2a). As for the data set containing EBV-transformed samples, we expect large-scale hypomethylation in the cancer samples and therefore we again consider *k*=100000 loci. The resulting ROC curves are shown in Figure 3a, and those for additional methods in Additional file 1: Figure S4a,b. Functional normalization and noob are best and do equally well. Again, the gold-standard set of probes that is used to measure performance in these ROC curves differs between normalization methods, and hence these ROC curves reflect the degree of consistency between experiments within each method.

To compare further the quality of the DMPs found by the different methods, we used an additional data set from TCGA where the same cancer was assayed with the Illumina 27k platform (see Materials and methods). We focused on the 25,978 CpG sites that were assayed on both platforms and asked about the size of the overlap for the top *k* DMPs. For the validation cohort, with the most unwanted variation, this is depicted in Figure 3b and Additional file 1: Figure S4c for additional methods; for the discovery cohort, with least unwanted variation, results are presented in Additional file 1: Figure S4. Functional normalization, together with noob, shows the best concordance in the presence of unwanted variation in the 450k data (the validation cohort) and is comparable to no normalization in the discovery cohort.

### Functional normalization preserves subtype heterogeneity in tumor samples

To measure how good our normalization method is at preserving biological variation among heterogeneous samples while removing technical biases, we use 192 acute myeloid leukemia samples (ACL) from TCGA for which every sample has been assayed on both the 27K and the 450K platforms (see Materials and methods). These two platforms assay 25,978 CpGs in common (but note the probe design changes between array types), and we can therefore assess the degree of agreement between measurements of the same sample on two different platforms, assayed at different time points. The 450k data appear to be affected by batch and dye bias; see Additional file 1: Figure S5.

**Figure 4:**
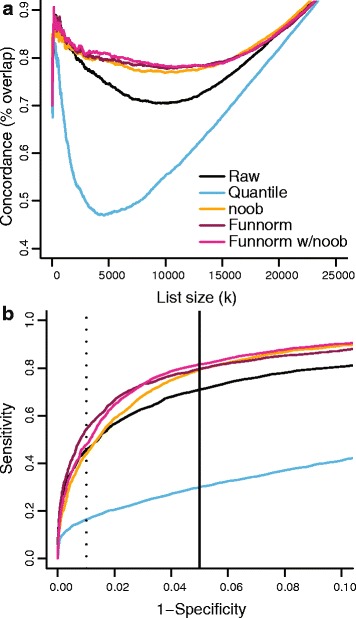
**Improvements in replication of tumor subtype heterogeneity.** In the AML data set from TCGA, the same samples have been assayed on 450k and 27k arrays. **(a)** Concordance plots between results from the 450k array and the 27k array. **(b)** ROC curves for the 450k data, using the results from the 27k data as gold standard. AML, acute myeloid leukemia; Funnorm, functional normalization; ROC, receiver operating characteristic; TCGA, The Cancer Genome Atlas.

Each sample was classified by TCGA according to the French-American-British (FAB) classification scheme [40], which proposes eight tumor subtypes, and methylation differences can be expected between the subtypes [41,42]. Using data from the 27k arrays, we identified the top *k* DMPs that distinguish the eight subtypes. In this case, we are assessing the agreement of subtype variability, as opposed to cancer–normal differences. The analysis of the 27k data uses unnormalized data but adjusts for sample batch in the model (see Materials and methods). Using data from the 450k arrays, we first processed the data using the relevant method, and next identified the top *k* DMPs between the eight subtypes. The analysis of the 450k data does not include a sample batch in the model, which allows us to see how well the different normalization methods remove technical artifacts introduced by batch differences. While both of the analyses are conducted on the full set of CpGs, we focus on the CpGs common between the two platforms and ask: ‘What is the degree of agreement between the top *k* DMPs identified using the two different platforms?’ Figure 4a shows that functional normalization and noob outperform both quantile normalization and raw data for all values of *k*, and functional normalization is marginally better than noob for some values of *k*. Additional file 1: Figure S6a shows the results for additional methods. We can also compare the two data sets using ROC curves, with the results from the 27k data as gold standard (Figure 4b and Additional file 1: Figure S6b). As for the DMPs for the 27k data, we used the 5,451 CpGs that demonstrate an estimated false discovery rate less than 5%. On the ROC curve functional normalization outperforms noob, quantile normalization and raw data for the full range of specificity.

### Replication between experiments with small changes

**Figure 5:**
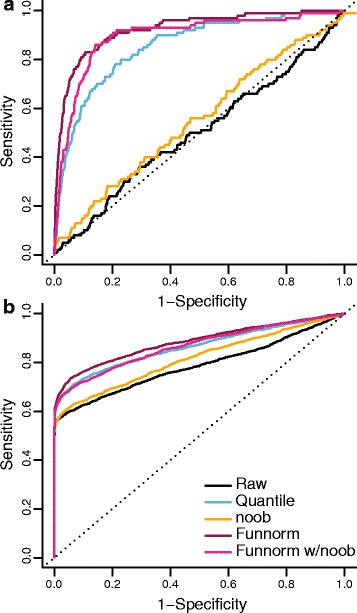
**Performance improvements on blood samples data set.** **(a)** ROC curve for replication of case–control differences between blood samples from colon cancer patients and blood samples from normal individuals, the Ontario-Blood data set. The validation data set was constructed to show an *in silico* batch effect. **(b)** ROC curve for identification of probes on the sex chromosomes for the Ontario-Sex data set. Sex is confounded by an *in silico* batch effect. Both evaluations show the good performance of functional normalization. Funnorm, functional normalization; ROC, receiver operating characteristic.

To measure the performance of functional normalization in a setting where there are no global changes in methylation, we used the Ontario-Blood data set, which has assays of lymphocytes from individuals with and without colon cancer. We expect a very small, if any, impact of colon cancer on the blood methylome. As above, we selected cases and controls to form discovery and validation cohorts, and we introduced *in silico* unwanted variation that confounds case–control differences in the validation data set only (see Materials and methods). The discovery and validation data sets contain, respectively, 283 and 339 samples. For *k*=100 loci, both functional and quantile normalization show good agreement between discovery and validation data sets, whereas noob and raw data show an agreement that is not better than a random selection of probes (Figure 5a, Additional file 1: Figure S7a).

### Functional normalization improves X and Y chromosome probe prediction in blood samples

As suggested previously [15], one can benchmark performance by identifying DMPs associated with sex. One copy of the X chromosome is inactivated and methylated in females, and the Y chromosome is absent. On the 450k array, 11,232 and 416 probes are annotated to be on the X and Y chromosomes, respectively. For this analysis it is sensible to remove regions of the autosomes that are similar to the sex chromosomes to avoid artificial false positives that are independent of the normalization step. We therefore remove a set of 30,969 probes that have been shown to cross-hybridize between genomic regions [43]. Because some genes have been shown to escape X inactivation [44], we only consider genes for which the X-inactivation status is known to ensure an unbiased sex prediction (see Materials and methods).

We introduced *in silico* unwanted variation by selecting 101 males and 105 females from different plates (see Materials and methods), thereby confounding plate with sex. Results show that functional normalization performs well (Figure 5b, Additional file 1: Figure S7b).

### Functional normalization reduces technical variability

**Figure 6:**
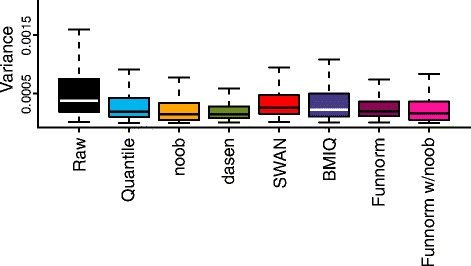
**Variance across technical triplicates.** Box plots of the probe-specific variances estimated across 19 individuals assayed in technical triplicates. All normalization methods improve upon raw data, and functional normalization performs well. funnorm, functional normalization; w, with.

From the Ontario-Replicates lymphocyte data set (see Materials and methods), we have 19 individuals assayed in technical triplicates dispersed among 51 different chips. To test the performance of each method to remove technical variation, we calculated the probe-specific variance within each triplicate, and averaged the variances across the 19 triplicates. Figure 6 presents box plots of these averaged probe variances of all methods. All normalization methods improve on raw data, and functional normalization is in the top three of the normalization methods. dasen, in particular, does well on this benchmark, which shows that improvements in reducing technical variation do not necessarily lead to similar improvements in the ability to replicate associations.

**Figure 7:**
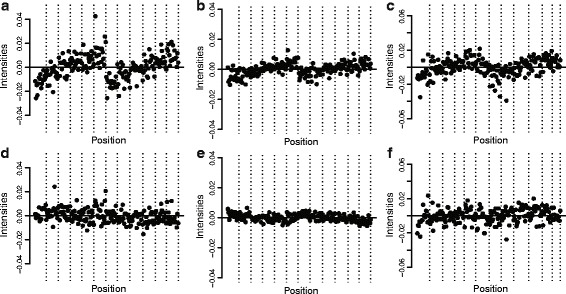
**Spatial location affects overall methylation.** Quantiles of the beta distributions adjusted for a slide effect. The 12 vertical stripes are ordered as rows 1 to 6 in column 1 followed by rows 1 to 6 in column 2. **(a)** 10th percentile for type II probes for the unnormalized AML data set. **(b)** 15th percentile for type I probes for the unnormalized AML data set. **(c)** 85th percentile for type II probes for the unnormalized Ontario-EBV data set. **(a–c)** show that the top of the slide has a different beta distribution from the bottom. **(d–f)** Like **(a–c)** but after functional normalization, which corrects this spatial artifact. AML, acute myeloid leukemia.

Each 450k array is part of a slide of 12 arrays, arranged in two columns and six rows (see Figure 7). Figure 7a–c shows an effect of column and row position on quantiles of the beta value distribution, across several slides. This effect is not present in all quantiles of the beta distribution, and it depends on the data set which quantiles are affected. Figure 7d–f shows that functional normalization corrects for this spatial artifact.

### Number of principal components

As described above, we recommend using functional normalization with the number of principal components set to *m*=2. Additional file 1: Figure S8 shows the impact of varying the number of principal components on various performance measures we have used throughout, and shows that *m*=2 is a good choice for the data sets we have analyzed. It is outperformed by *m*=6 in the analysis of the KIRC data and by *m*=3 in the analysis of the AML data, but these choices perform worse in the analysis of the Ontario-EBV data. While *m*=2 is a good choice across data sets, we leave *m* to be a user-settable parameter in the implementation of the algorithm. This analysis assumes we use the same *m* for the analysis of both the discovery and validation data sets. We do this to prevent overfitting and to construct an algorithm with no user input. It is possible to obtain better ROC curves by letting the choice of *m* vary between discovery and validation, because one data set is confounded by batch and the other is not.

### Comparison to batch effect removal tools

Batch effects are often considered to be unwanted variation remaining after an unsupervised normalization. In the previous assessments, we have comprehensively compared functional normalization to existing normalization methods and have shown great performance in the presence of unwanted variation. While functional normalization is an unsupervised normalization procedure, we were interested in comparing its performance to supervised normalization methods, such as SVA [25,26], RUV [28] and ComBat [27]. We adapted RUV to the 450k array (see Materials and methods) and used reference implementations for the other two methods [45].

**Figure 8:**
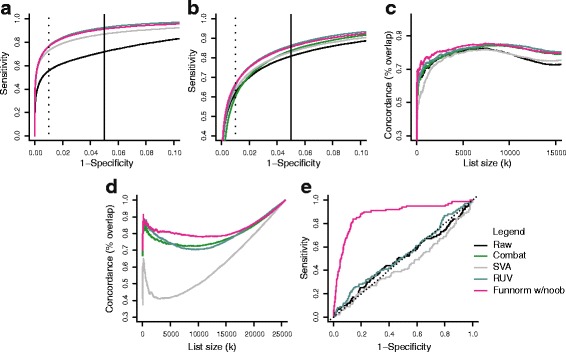
**Comparison to batch effect removal tools SVA, RUV and ComBat.** **(a)** Like Figure 2a, an ROC curve for the Ontario-EBV data set. **(b)** Like Figure 3a, an ROC curve for the TCGA-KIRC data set. **(c)** Like Figure 3b, a concordance curve between the validation cohort from 450k data and the 27k data for the TCGA-KIRC data set. **(d)** Like Figure 4a, concordance plots between results from the 450k array and the 27k array for the TCGA-AML data set. **(e)** Like Figure 5a, an ROC curve for the Ontario-Blood data set. AML, acute myeloid leukemia; EBV, Epstein–Barr virus; Funnorm, functional normalization; ROC, receiver operating characteristic.

We applied these three batch removal tools to all data sets analyzed previously. We let SVA estimate the number of surrogate variables, and allowed this estimation to be done separately on the discovery and the validation data sets, which allowed for the best possible performance by the algorithm. For RUV, we selected negative control probes on the array as negative genes and probes mapping to the X and Y chromosomes as positive genes in the language of RUV (see Materials and methods for details). These negative and positive genes were used to select the number of unwanted factors, as per the recommendations in Gagnon-Bartsch and Speed [28]. Figure 8 compares the three methods to functional normalization and raw data for our evaluation data sets. The three methods have the greatest difficulty with the TCGA-AML and the Ontario-Blood data sets compared to functional normalization. Functional normalization is still a top contender for the Ontario-EBV and the TCGA-KIRC data sets, although RUV does outperform functional normalization slightly on Ontario-EBV. This shows that unsupervised functional normalization outperforms these three supervised normalization methods on multiple data sets.

### The effect of normalization strategy on effect size estimates

**Figure 9:**
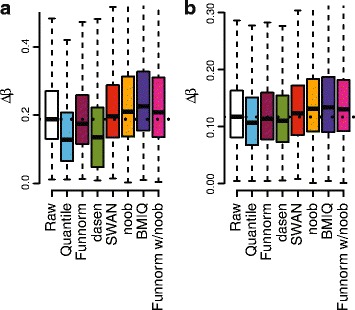
**Effect size of the top replicated loci.** Box plots represent the effect sizes for the top *k* loci from the discovery cohort that are replicated in the validation cohort. The effect size is measured as the difference on the beta value scale between the two treatment group means. **(a)** Box plots for the top *k*=100000 loci replicated in the Ontario-EBV data set. **(b)** Box plots for the top *k*=100000 loci replicated in the TCGA-KIRC data set. EBV, Epstein–Barr virus; Funnorm, functional normalization; w, with.

To assess the impact of normalization on the estimated effect sizes, we computed estimated methylation differences on the Beta scale between cases and controls for the Ontario-EBV and KIRC data sets. Figure 9 shows the distribution of effect sizes for the top loci in the discovery data sets that are replicated in the validation data sets. The impact of the normalization method on these distributions depends on the data set.

### The performance of functional normalization for smaller sample sizes

**Figure 10:**
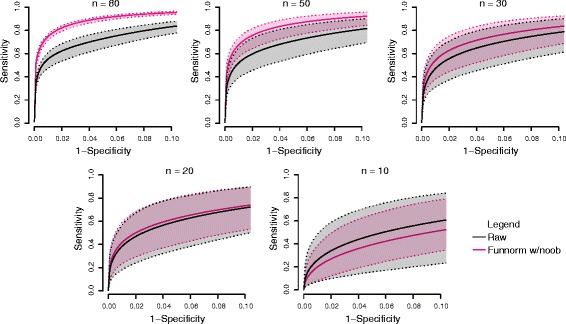
**Sample size simulation for the Ontario-EBV data set.** Partial discovery–validation ROC curves for the Ontario-EBV data set similar to Figure 2a but for random subsamples of different sizes *n*=10,20,30,50 and 80. Each solid line represents the mean of the ROC results for *B*=100 subsamples of size *n*. The dotted lines represent the 0.025 and 0.975 percentiles. EBV, Epstein–Barr virus; Funnorm, functional normalization; ROC, receiver operating characteristic.

To assess the performance of functional normalization with small sample sizes, we repeated the analysis of the Ontario-EBV data set with different sample sizes by randomly subsampling an equal number of arrays from the two treatment groups multiple times. For instance, for sample size *n*=30, we randomly drew 15 lymphocyte samples and 15 EBV-transformed samples. We repeated the subsampling *B*=100 times and calculated 100 discovery–validation ROC curves. Figure 10 shows the mean ROC curves together with the 0.025 and 0.975 percentiles for both the raw data and the data normalized with functional normalization with noob, for different sample sizes. At a sample size of 20, functional normalization very slightly outperforms raw data, and functional normalization improves on raw data with sample sizes *n*≥30.

## Conclusions

We have presented functional normalization, an extension of quantile normalization, and have adapted this method to Illumina 450k methylation microarrays. We have shown that this method is especially valuable for normalizing large-scale studies where we expect substantial global differences in methylation, such as in cancer studies or when comparing between tissues, and when the goal is to perform inference at the probe level. Although an unsupervised normalization method, functional normalization is robust in the presence of a batch effect, and performs better than the three batch removal tools, ComBat, SVA and RUV, on our assessment data sets. This method fills a critical void in the analysis of DNA methylation arrays.

We have evaluated the performance of our method on a number of large-scale cancer studies. Critically, we define a successful normalization strategy as one that enhances the ability to detect associations between methylation levels and phenotypes of interest reliably across multiple experiments. Various other metrics for assessing the performance of normalization methods have been used in the literature on preprocessing methods for Illumina 450k arrays. These metrics include assessing variability between technical replicates [13,14,16,17,46], and comparing methylation levels to an external gold standard measurement, such as bisulfite sequencing [11,14,17]. We argue that a method that yields unbiased and precise estimates of methylation in a single sample does not necessarily lead to improvements in estimating the differences between samples, yet the latter is the relevant end goal for any scientific investigation. This is a consequence of the well-known bias-variance trade-off [47]. An example of this trade-off for microarray normalization is the performance of the RMA method [48] for analysis of Affymetrix gene expression microarrays. This method introduces bias into the estimation of fold-changes for differentially expressed genes; however, this bias is offset by a massive reduction in variance for non-differentially expressed genes, leading to the method’s proven performance. Regarding reducing technical variation, we show in Figure 6 that methods that show the greatest reduction in technical variation do not necessarily have the best ability to replicate findings, and caution the use of this assessment for normalization performance.

In our comparisons, we have separately normalized the discovery and the validation data sets, to mimic replication across different experiments. We have shown that functional normalization was always amongst the top performing methods, whereas other normalization methods tended to perform well on some, but not all, of our test data sets. As suggested by Dedeurwaerder *et al.* [17], our benchmarks showed the importance of comparing performance to raw data, which outperformed (using our metrics) some of the existing normalization methods. For several data sets, we have observed that the within-array normalization methods SWAN and BMIQ had very modest performance compared to raw data and between-array normalization methods. This suggests that using within-array normalization methods do not lead to improvements in the ability to replicate findings between experiments.

Our closest competitor is noob [16], which includes both a background correction and a dye-bias equalization. We outperformed noob substantially for the Ontario-Blood and Ontario-Sex data sets and we performed slightly better on the TCGA-AML data set. The best performance was obtained by using functional normalization after the noob procedure.

Our method relies on the fact that control probes carry information about unwanted variation from a technical source. This idea was also used by Gagnon-Bartsch and Speed [28] to design the batch removal tool RUV. As discussed in the Results section, the RUV method is tightly integrated with a specific statistical model, requires the specification of the experimental design, and cannot readily accommodate regional methods [12,36,37] nor clustering. In contrast, functional normalization is completely unsupervised and returns a corrected data matrix, which can be used as input into any type of downstream analysis, such as clustering or regional methods. Batch effects are often considered to be unwanted variation remaining after an unsupervised normalization, and we conclude that functional normalization removes a greater amount of unwanted variation in the preprocessing step. It is interesting that this is achieved merely by correcting the marginal densities.

However, control probes cannot measure unwanted variation arising from factors representing variation present in the samples themselves, such as cell-type heterogeneity, which is known to be an important confounder in methylation studies of samples containing mixtures of cell types [33]. This is an example of unwanted variation from a biological, as opposed to technical, source. Cell-type heterogeneity is a particular challenge in EWAS studies of whole blood, but this has to be addressed by other tools and approaches.

Surprisingly, we showed that functional normalization improved on the batch removal tools, ComBat, SVA and RUV, applied to raw data, in the data sets we have assessed. It is a very strong result that an unsupervised normalization method improves on supervised normalization procedures, which require the specification of the comparison of interest.

While we have shown that functional normalization performed well in the presence of unwanted variation, we still recommend that any large-scale study considers the application of batch removal tools, such as SVA [25,26], ComBat [27] and RUV [28], after using functional normalization, due to their proven performance and their potential for removing unwanted variation that cannot be measured by control probes. As an example, Jaffe and Irizarry [33] discuss the use of such tools to control for cell-type heterogeneity.

The analysis of the Ontario-Blood data set suggests that functional normalization has potential to improve the analysis in a standard EWAS setting, in which only a small number of differentially methylated loci are expected. However, if only very few probes are expected to change, and if those changes are small, it becomes difficult to evaluate the performance of our normalization method using our criteria of successful replication.

The main ideas of functional normalization can readily be applied to other microarray platforms, including gene expression and miRNA arrays, provided that the platform of interest contains a suitable set of control probes. We expect the method to be particularly useful when applied to data with large anticipated differences between samples.

## Materials and methods

### Infinium HumanMethylation450 BeadChip

We use the following terminology, consistent with the minfi package [12]. The 450k array is available as slides consisting of 12 arrays. These arrays are arranged in a six rows by two columns layout. The scanner can process up to eight slides in a single plate. We use the standard formula *β*=*M*/(*M*+*U*+100) for estimating the percentage methylation given (un)methylation intensities *U* and *M*.

Functional normalization uses information from the 848 control probes on the 450k array, as well as the out-of-band probes discussed in Triche *et al.* [16]. These control probes are not part of the standard output from GenomeStudio, the default Illumina software. Instead we use the IDAT files from the scanner together with the open source illuminaio [49] package to access the full data from the IDAT files. This step is implemented in minfi [12]. While not part of the standard output from GenomeStudio, it is possible to access the control probe measures within this software by accessing the Control Probe Profile.

### Control probe summaries

We transform the 848 control probes, as well as the out-of-band probes [16] into 42 summary measures. The control probes contribute 38 of these 42 measures and the out-of-band probes contribute four. An example of a control probe summary is the mapping of 61 ‘C’ normalization probes to a single summary value, their mean. The out-of-band probes are the intensities of the type I probes measured in the opposite color channel from the probe design. For the 450k platform, this means 92,596 green intensities, and 178,406 red intensities that can be used to estimate background intensity, and we summarize these values into four summary measures. A full description of how the control probes and the out-of-band probes are transformed into the summary control measures is given in Additional file 1: Supplementary material.

### Functional normalization: the general framework

Functional normalization extends the idea of quantile normalization by adjusting for known covariates measuring unwanted variation. In this section, we present a general model that is not specific to methylation data. The adaptation of this general model to the 450k data is discussed in the next section. The general model is as follows. Consider **Y**_1_,…,**Y**_*n*_ high-dimensional vectors each associated with a set of scalar covariates *Z*_*i*,*j*_ with *i*=1,…,*n* indexing samples and *j*=1,…,*m* indexing covariates. Ideally these known covariates are associated with unwanted variation and unassociated with biological variation; functional normalization attempts to remove their influence. For each high-dimensional observation **Y**_*i*_, we form the empirical quantile function for its marginal distribution, and denote it by $q^{\text {emp}}_{i}$. Quantile functions are defined on the unit interval and we use the variable *r*∈[0,1] to evaluate them pointwise, like $q^{\text {emp}}_{i}(r)$. We assume the following model in pointwise form

(1) $$ q^{\text{emp}}_{i}(r) = \alpha(r)+ \sum\limits_{j=1}^{m} Z_{i,j}\beta_{j}(r) + \epsilon_{i}(r),  $$

which has the functional form

(2) $$ q^{\text{emp}}_{i} = \alpha + \sum\limits_{j=1}^{m} Z_{i,j}\beta_{j} + \epsilon_{i}  $$

The parameter function *α* is the mean of the quantile functions across all samples, *β*_*j*_ are the coefficient functions associated with the covariates and *ε*_*i*_ are the error functions, which are assumed to be independent and centered around 0.

In this model, the term

(3) $$ \sum\limits_{j=1}^{m} Z_{i,j}\beta_{j}  $$

represents variation in the quantile functions explained by the covariates. By specifying known covariates that measure unwanted variation and that are not associated with a biological signal, functional normalization removes unwanted variation by regressing out the latter term. An example of a known covariate could be processing batch. In a good experimental design, processing batch will not be associated with a biological signal.

In particular, assuming we have obtained estimates $\hat {\beta }_{j}$ for *j*=1,…,*m*, we form the functional normalized quantiles by

(4) $$ q^{\text{Funnorm}}_{i}(r) = q^{\text{emp}}_{i}(r)- \sum\limits_{j=1}^{m} Z_{i,j}\hat{\beta}_{j}(r)  $$

We then transform **Y**_*i*_ into the functional normalized quantity $\tilde {\mathbf {Y}}_{i}$ using the formula

(5) $$ \tilde{\mathbf{Y}}_{i} = q^{\text{Funnorm}}_{i} \left(\left(q^{\text{emp}}_{i}\right)^{-1} (\mathbf{Y}_{i}) \right)  $$

This ensures that the marginal distribution of $\tilde {\mathbf {Y}}_{i}$ has $q^{\text {Funnorm}}_{i}$ as its quantile function.

We now describe how to obtain estimates $\hat {\beta }_{j}$ for *j*=1,…,*m*. Our model 1 is an example of function-on-scalar regression, described in [50]. The literature on function-on-scalar regression makes assumptions about the smoothness of the coefficient functions and uses a penalized framework because the observations appear noisy and non-smooth. In contrast, because our observations **Y**_*i*_ are high dimensional and continuous, the jumps of the empirical quantile functions are very small. This allows us to circumvent the smoothing approach used in traditional function-on-scalar regression. We use a dense grid of *H* equidistant points between 0 and 1, and we assume that *H* is much smaller than the dimension of **Y**_*i*_. On this grid, model 1 reduces pointwise to a standard linear model. Because the empirical quantile functions *q*^emp^(*r*) have very small jumps, the parameter estimates of these linear models vary little between two neighboring grid points. This allows us to use *H* standard linear model fits to compute estimates $\hat {\alpha }(h)$ and $\hat {\beta }_{j}(h)$, *j*=1,…,*m*, with *h* being on the dense grid {*h*∈*d*/*H*:*d*=0,1,…,*H*}. We next form estimates $\hat {\alpha }(r)$ and $\hat {\beta }_{j}(r)$, *j*=1,…,*m*, for any *r*∈[0,1] by linear interpolation. This is much faster than the penalized function-on-scalar regression available through the refund package [51].

Importantly, in this framework, using a saturated model in which all the variation (other than the mean) is explained by the covariates results in removing all variation and is equivalent to quantile normalization. In our notation, quantile-normalized quantile functions are

(6) $$ q_{i}^{\text{quantile}}(r) = \hat{\alpha}(r)  $$

where $\hat {\alpha }$ is the mean of the empirical quantile functions. This corresponds to the maximum variation that can be removed in our model. In contrast, including no covariates makes the model comparable to no normalization at all. By choosing covariates that only measure unwanted technical variation, functional normalization will only remove the variation explained by these technical measurements and will leave biological variation intact. Functional normalization allows a sensible trade-off between not removing any technical variation at all (no normalization) and removing too much variation, including global biological variation, as can occur in quantile normalization.

### Functional normalization for 450k arrays

We apply the functional normalization model to the methylated (*M*) and unmethylated (*U*) channels separately. Since we expect the relationship between the methylation values and the control probes to differ between type I and type II probes, functional normalization is also applied separately by probe type to obtain more representative quantile distributions. We address the mapping of probes to the sex chromosomes separately; see below. This results in four separate applications of functional normalization, using the exact same covariate matrix, with more than 100,000 probes in each normalization fit. For functional normalization, we pick *H*=500 equidistant points (see notation in previous section). As covariates, we use the first *m*=2 principal components of the summary control measures as described above. We do this because the control probes are not intended to measure a biological signal since they are not designed to hybridize to genomic DNA. Our choice of *m*=2 is based on empirical observations on several data sets.

Following the ideas from quantile normalization for 450k arrays [11,12], we normalize the mapping of probes to the sex chromosomes (11,232 and 416 probes for the X and Y chromosomes, respectively) separately from the autosomal probes. For each of the two sex chromosomes, we normalize males and females separately. For the X chromosome, we use functional normalization, and for the Y chromosome, we use quantile normalization, since the small number of probes on this chromosome violates the assumptions of functional normalization, which results in instability.

Functional normalization only removes variation in the marginal distributions of the two methylation channels associated with control probes. This preserves any biological global methylation difference between samples. We have found (see Results) that we get slightly better performance for functional normalization if we apply it to data that have been background corrected with noob [16].

### Data

**The Ontario study.** The Ontario study consists of samples from 2,200 individuals from the Ontario Familial Colon Cancer Registry [52] who had previously been genotyped in a case–control study of colorectal cancer in Ontario [53]. The majority of these samples are lymphocytes derived from whole blood. We use various subsets of this data set for different purposes.

Biospecimens and data collected from study participants were obtained with written informed consent and approval from the University of Toronto Office of Research Ethics (Protocol Reference 23212), in compliance with the WMA Declaration of Helsinki – Ethical Principles for Medical Research Involving Human Subjects.

**The Ontario-EBV data set.** Lymphocyte samples from 100 individuals from the Ontario study were transformed into immortal lymphoblastoid cell lines using the EBV transformation. We divided the 100 EBV-transformed samples into two equal-sized data sets (discovery and validation). For the discovery data set, we matched the 50 EBV-transformed samples to 50 other lymphocyte samples assayed on the same plates. For the validation data set, we matched the 50 EBV-transformed samples to 50 other lymphocyte samples assayed on different plates.

**The Ontario-Blood data set.** From the Ontario study, we first created a discovery–validation design where we expect only a small number of loci to be differentially methylated. For the discovery data set, we selected all cases and controls on three plates that showed little evidence of plate effects among the control probes, which yielded a total of 52 cases and 231 controls. For the validation data set, we selected four plates where the control probes did show evidence of a plate effect and then selected cases and controls from separate plates, to maximize the confounding effect of plate. This yielded a total of 175 cases and 163 controls.

**The Ontario-Sex data set.** Among ten plates for which the control probes demonstrated differences in distribution depending on plate, we selected 101 males from a set of five plates and 105 females from another set of five plates, attempting to maximize the confounding effect of batch on sex.

**The Ontario-Replicates data set.** Amongst the lymphocyte samples from the Ontario study, 19 samples have been assayed three times each. One replicate is a hybridization replicate and the other replicate is a bisulfite conversion replicate. The 57 samples have been assayed on 51 different slides across 11 different plates.

**The TCGA-KIRC data sets.** From TCGA, we have access to KIRC and normal samples, assayed on two different methylation platforms. We use the level 1 data, contained in IDAT files. For the 450k platform, TCGA has assayed 300 tumor samples and 160 normal samples. For the discovery set, we select 65 tumor samples and 65 matched normal samples from slides showing little variation in the control probes. These 130 samples were assayed on three plates. For the validation data set, we select the remaining 95 normal samples together with all 157 cancer samples that were part of the same TCGA batches as the 95 normal samples. These samples were spread over all nine plates, therefore maximizing potential batch effects. For the 27k platform, TCGA has assayed 219 tumor samples and 199 normal samples. There is no overlap between the individuals assayed on the 450k platform and the individuals assayed on the 27k platform.

**The TCGA-AML data sets.** Also from TCGA, we used data from 194 AML samples, where each sample was assayed twice: first on the 27K Illumina array and subsequently on the 450K array. Every sample but two has been classified according to the FAB subtype classification scheme [40], which classifies tumors into one of eight subtypes. The two unclassified samples were removed post-normalization. We use the data known as level 1, which is contained in IDAT files.

**Whole-genome bisulfite sequencing (WGBS) EBV data.** Hypomethylated blocks and small DMRs between transformed and quiescent cells were obtained from a previous study [38]. Only blocks and DMRs with a family-wise error rate equal to 0 were retained (see the reference). A total of 228,696 probes on the 450K array overlap with the blocks and DMRs.

### Data availability

The Ontario methylation data have been deposited in dbGAP under accession number [phs000779.v1.p1]. These data were available to researchers under the following constraints: (1) the use of the data is limited to research on cancer, (2) the researchers have local Institutional Review Board approval and (3) the researchers have the approval of either Colon Cancer Family Registries [54] or Mount Sinai Hospital (Toronto) Research Ethics Board. The TCGA data (KIRC and AML) are available through the TCGA Data Portal [55]. The WGBS EBV data is available through the Gene Expression Omnibus of the National Center for Biotechnology Information under the accession number [GEO:GSE49629]. Our method is available as the preprocessFunnorm function in the minfi package through the Bioconductor project [56]. The code in this package is licensed under the open-source license Artistic-2.0.

### Data processing

Data were available in the form of IDAT files from the various experiments (see above). We used minfi [12] and illuminaio [49] to parse the data and used the various normalization routines in their reference implementations (see below).

We performed the following quality control on all data sets. As recommended in Touleimat and Tost [11], for each sample we computed the percentage of loci with a detection *P* value greater than 0.01, with the intention of excluding a sample if the percentage was higher than 10%. We used the minfi [12] implementation of the detection *P* value. We also used additional quality control measures [12] and we interactively examined the arrays using the shinyMethyl package [57]; all arrays in all data sets passed our quality control.

We performed the following filtering of loci, after normalization. We removed 17,302 loci that contain a SNP with an annotated minor allele frequency greater than or equal to 1% in the CpG site itself or in the single-base extension site. We used the UCSC Common SNPs table based on dbSNP 137; this table is included in the minfi package. We removed 29,233 loci that have been shown to cross-hybridize to multiple genomic locations [43]. The total number of loci removed is 46,535, i.e. 9.6% of the array. We chose to remove these loci post-normalization as done previously [16,58], reasoning that while these probes may lead to spurious associations, we believe they are still subject to technical variation and should therefore contain information useful for normalization.

### Comparison to normalization methods

We have compared functional normalization to the most popular normalization methods used for the 450k array. This includes the following between-array normalization methods: (1) quantile: stratified quantile normalization as proposed by Touleimat *et al.* [11] and implemented in minfi [12], (2) dasen: background adjustment and between-sample quantile normalization of *M* and *U* separately [15] and (3) noob: a background adjustment model using the out-of-band control probes followed by a dye bias correction [16], implemented in the methylumi package. We also consider two within-array normalization methods: (4) SWAN [13] and (5) BMIQ [14]. Finally, we consider (6) raw data: no normalization, i.e., we only matched up the red and the green channels with the relevant probes according to the array design (specifically, it is the output of the preprocessRaw function in minfi).

In its current implementation, noob yielded missing values for at most a couple of thousand loci (less than 1%) per array. This is based on excluding loci below an array-specific detection limit. We have discarded those loci from our performance measures, but only for the noob performance measures. In its current implementation, BMIQ produced missing values for all type II probes in five samples for the TCGA-AML data set. We have excluded these samples for our performance measures, but only for our BMIQ performance measures.

For clarity, in the figures we focus on the top-performing methods which are raw data, and quantile and noob normalization. The assessments of the other methods, dasen, BMIQ and SWAN, are available in Additional file 1: Supplementary Materials.

### Comparison to SVA

We used the reference implementation of SVA in the sva package [45]. We applied SVA to the *M* values obtained from the raw data. Surrogate variables were estimated using the iteratively re-weighted SVA algorithm [26], and were estimated separately for the discovery and validation cohorts. In the analysis of the Ontario-EBV data set, SVA found 21 and 23 surrogate variables, respectively, for the discovery and the validation cohorts. In the analysis of the Ontario-Blood data set, SVA found 18 and 21 surrogate variables, respectively, for the discovery and the validation cohorts. In the analysis of the TCGA-KIRC data set, SVA found 29 and 32 surrogate variables, respectively, for the discovery and the validation cohorts. In the analysis of the TCGA-AML data set, SVA found 24 surrogate variables.

### Comparison to RUV

The RUV-2 method was originally developed for gene expression microarrays [28]. The method involves a number of domain-specific choices. To our knowledge, there is no publicly available adaption of RUV-2 to the 450k platform, so we adapted RUV-2 to the 450k array. The core of the method is implemented in software available from a personal website [59]. As negative genes (genes not associated with the biological treatment group), we selected the raw intensities in the green and red channels of the 614 internal negative control probes available on the 450k array.

To determine the number *k* of factors to remove (see Gagnon-Bartsch and Speed [28] for details of this parameter), we followed the approach described in [28]. First, for each value *k*=0,1,…,40, we performed a differential analysis with respect to sex. Second, we considered as positive controls the probes that are known to undergo X inactivation (see section Sex validation analysis) and probes mapping to the Y chromosome. Third, for the top ranked *m*=25000, 50,000 and 100,000 probes, we counted how many of the positive control probes are present in the list. Finally, we picked the value of *k* for which these counts are maximized. The different tuning plots are presented in Additional file 1: Figure S9. The optimal *k* was 14 and 11 for the discovery and the validation cohorts of the Ontario-EBV data set, respectively. In the analysis of the Ontario-Blood data set, the optimal *k* was 0 and 3, respectively, for the discovery and the validation cohorts. In the analysis of the TCGA-KIRC data set, the optimal *k* was 36 and 5, respectively, for the discovery and the validation cohorts. In the analysis of the TCGA-AML data set, *k* was selected to be 0 (which is equivalent to raw data).

### Comparison to ComBat

We used the reference implementation of ComBat in the sva package [45]. Because ComBat cannot be applied to data sets for which the phenotype of interest is perfectly confounded with the batch variable, we could only run ComBat for the AML and KIRC data sets.

### Identification of differentially methylated positions

To identify DMPs, we used *F*-statistics from a linear model of the beta values from the array. The linear model was applied on a probe-by-probe basis. In most cases, the model included case/control status as a factor. In the 27K data, we adjusted for batch by including a plate indicator (given by TCGA) in the model.

### Discovery–validation comparisons

To measure the consistency of each normalization method at finding true DMPs, we compared results obtained on a discovery–validation split of a large data set. Comparing results between two different subsets of a large data set is an established idea and has been applied to the context of 450k normalization [14,46]. We extended this basic idea in a novel way by introducing an *in silico* confounding of treatment (case/control status) by batch effects as follows. In a first step, we selected a set of samples to be the discovery cohort, by choosing samples where the treatment variable is not visibly confounded by plate effects. Then the validation step is achieved by selecting samples demonstrating strong potential for treatment confounding by batch, for example by choosing samples from different plates (see descriptions of the data). The extent to which it is possible to introduce such a confounding depends on the data set. In contrast to earlier work [46], we normalized the discovery and the validation cohorts separately, to mimic an independent replication experiment more realistically. The idea of creating *in silico* confounding between batch and treatment has been previously explored in the context of genomic prediction [39].

We quantified the agreement between validation and discovery in two ways: by an ROC curve and a concordance curve. For the ROC curve, we used the discovery cohort as the gold standard. Because the validation cohort is affected by a batch effect, a normalization method that is robust to batch effects will show better performance on the ROC curve. Making this ROC curve required us to choose a set of DMPs for the discovery cohort. The advantage of the ROC curve is that the plot displays immediately interpretable quantities, such as specificity and sensitivity.

For the concordance curve, we compared the top *k* DMPs from the discovery and the validation sets, and displayed the percentage of the overlap for each *k*. These curves do not require us to select a set of DMPs for the discovery cohort. Note that these curves have been previously used in the context of multiple-laboratory comparison of microarray data [60].

### Sex validation analysis

On the 450k array, 11,232 and 416 probes map to the X and Y chromosomes, respectively. Because some genes have been shown to escape X inactivation [44], we only considered genes for which the X-inactivation status is known to ensure an unbiased sex prediction. From [44], 1,678 probes undergo X-inactivation, 140 probes escape X-inactivation, and 9,414 probes have either variable or unknown status.

For the ROC curves, we defined the true positives to be the 1,678 probes undergoing X-inactivation and the probes mapping to the Y chromosome (416 probes); by removing the probes that have been shown to cross-hybridize [43], we were left with 1,877 probes. For the true negatives, we considered the 140 probes escaping X-inactivation and the autosomal probes that do not cross-hybridize. The rest of the probes were removed from the analysis.

### Sample size simulation

To assess the performance of functional normalization for different small sample sizes, we devised the following simulation scheme for the Ontario-EBV data set. First, we kept the discovery data set intact to ensure a reasonable gold standard in the discovery–validation ROC curves; we only simulated different sample sizes for the validation subset. For sample sizes *n*=10,20,30,50 and 80, we randomly chose half of the samples from the EBV-transformed samples, and the other half from the lymphocyte samples. For instance, for *n*=10 samples, we randomly picked five samples from each of the treatment groups. We repeated this subsampling *B*=100 times, which generated 100 discovery–validation ROC curves for each *n*. For a fixed *n*, we considered the mean of the *B*=100 ROC curves as well as the 0.025 and 0.975 quantiles to mimic a 95% confidence interval.

### Reproducibility

A machine-readable document detailing our analyses is available at GitHub [61].

## Additional file

Additional file 1 **Supplementary information.** Supplementary Figures S1–S9 and supplementary material with a description of how control probes are treated.

## Abbreviations

AML: acute myeloid leukemia
BMIQ: beta-mixture quantile method
DMP: differentially methylation position
DMR: differentially methylation region
EBV: Epstein–
Barr virus; EWAS: epigenome-wise association studies
FAB: French-American-British classification
KIRC: kidney clear-cell carcinoma
miRNA: micro-RNA
ROC: receiver operating characteristic
RMA: robust multi-array average
RUV: remove unwanted variation
SNM: supervised normalization of microarrays
SNP: single nucleotide polymorphism
SVA: surrogate variable analysis
SWAN: subset-quantile within array normalization
TCGA: The Cancer Genome Atlas
VSN: variance-stabilizing normalization
WGBS: whole-genome bisulfite sequencing
