# Supplementary material for: Functional normalization of 450k methylation array data improves replication in large cancer studies
Source: Genome Biol. 2014 Dec 3;15(11):503. doi: 10.1186/s13059-014-0503-2 (PMC4283580; doi:10.1186/s13059-014-0503-2)
Supplement: Supplementary file 1 — Supplementary information. Supplementary Figures S1–S9 and supplementary material with a description of how control probes are treated. [file 13059_2014_503_MOESM1_ESM.pdf]

Supplementary Information for Functional normalization of 450k methylation array data improves replication in large cancer studies

Jean-Philippe Fortin[1], Aurélie Labbe[2,3,4], Mathieu Lemire[5], Brent W. Zanke[6], Thomas J. Hudson[5,7], Elana J. Fertig[8], Celia M.T. Greenwood[2,9,10], and Kasper D. Hansen[1,11,*]

[1]Department of Biostatistics, Johns Hopkins Bloomberg School of Public Health
[2]Department of Epidemiology, Biostatistics and Occupational Health, McGill University
[3]Douglas Mental Health University Institute, McGill University
[4]Department of Psychiatry, McGill University
[5]Ontario Institute of Cancer Research
[6]Clinical Epidemiology Program, Ottawa Hospital Research Institute
[7]Departments of Molecular Genetics and Medical Biophysics, University of Toronto
[8]Department of Oncology, Sidney Kimmel Cancer Center, Johns Hopkins School of Medicine
[9]Lady Davis Institute for Medical Research, Jewish General Hospital Montreal
[10]Department of Oncology, McGill University
[11]McKusick-Nathans Institute of Genetic Medicine, Johns Hopkins School of Medicine

[*]To whom correspondence should be addressed. Email: khansen@jhsph.edu

Supplementary Figures

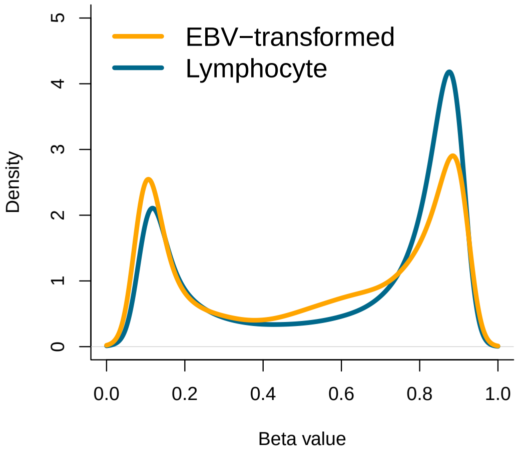

(a)

Supplementary Figure S1. Global changes in DNA methylation for the EBV dataset. (a) The average density of unnormalized beta values across both EBV transformed lymphocytes and normal lymphocytes, showing global hypomethylation caused by EBV transformation.

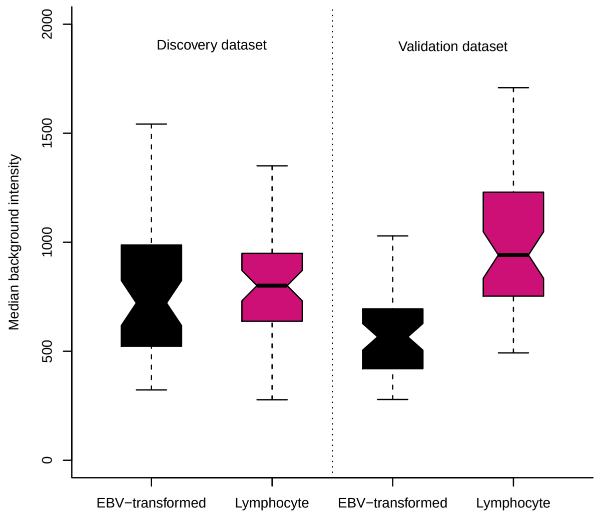

(a)

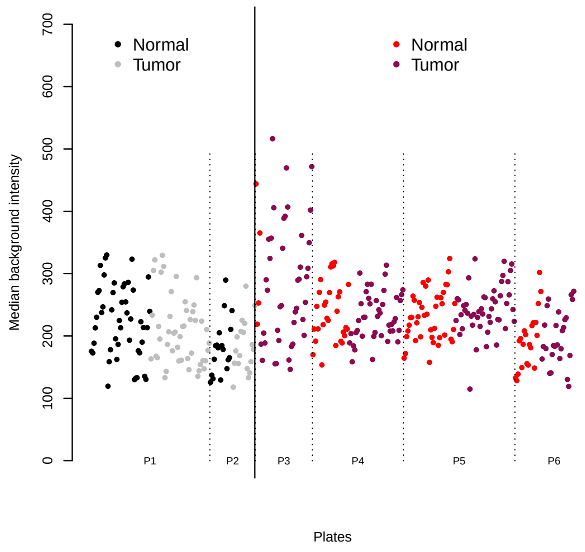

(b)

Supplementary Figure S2. Illustration of *in silico* batch effects. (a) Distribution of background intensity for the Ontario-EBV dataset, showing an *in silico* introduced difference in the validation dataset. (b) Distribution of background intensity for the TCGA KIRC dataset, showing an *in silico* introduced difference in variation in the validation dataset.

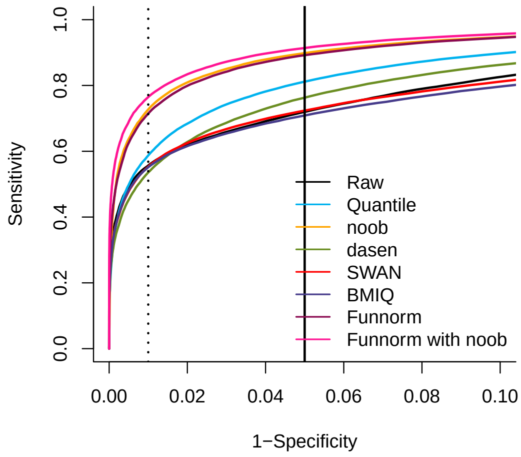

(a)

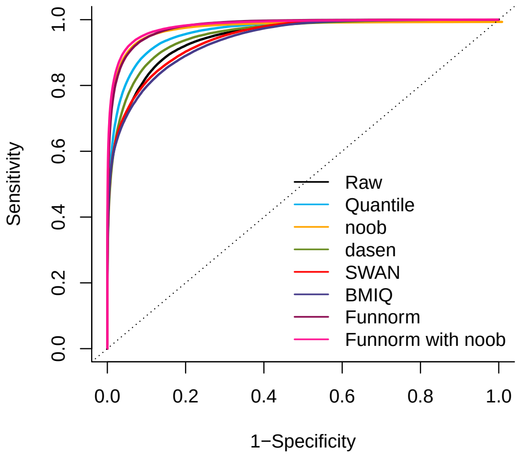

(b)

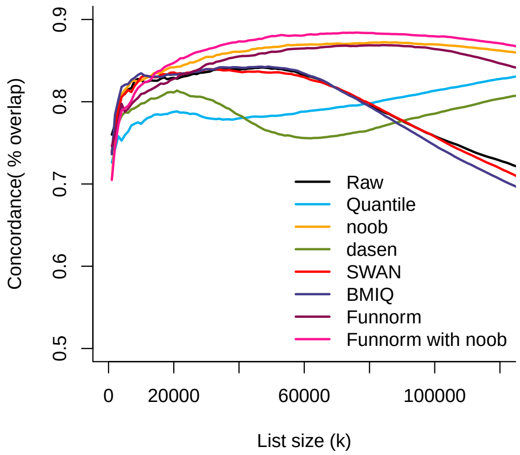

(c)

Supplementary Figure S3. Improvements in replication for the EBV dataset, all methods. (a) Like Figure 2b, but for all examined normalization methods. (b) Like (a) but for the full range of specificity. (c) Like Figure 2c but for all examined normalization methods.

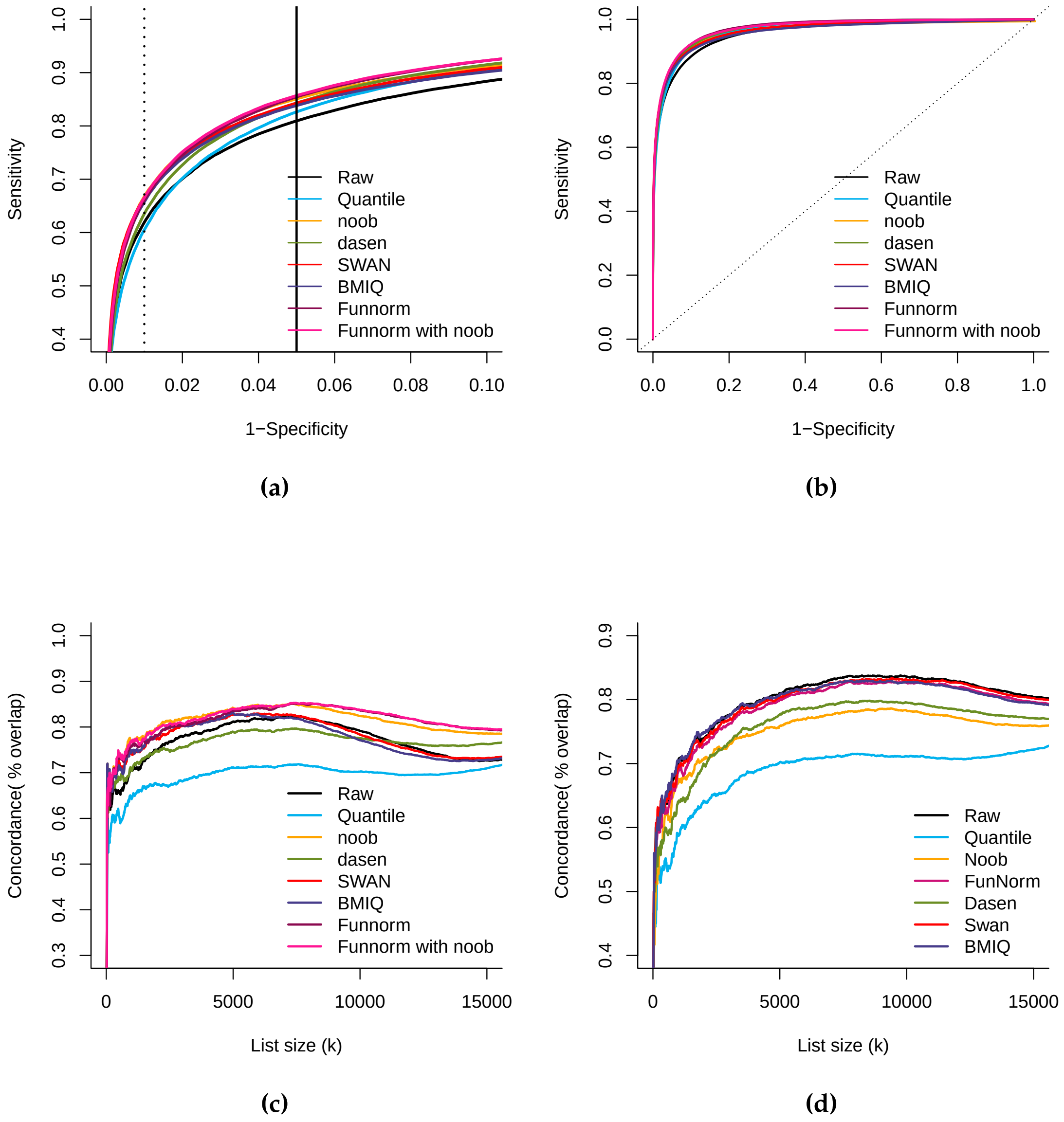

Supplementary Figure S4. Improvements in replication for the TCGA KIRC dataset, all methods. (a) Like Figure 3a but for all normalization methods we assess. (b) Like (a) but for the full range of specificity. (c) Like Figure 3b but for all normalization methods we assess. (d) Like (c) but comparing to the discovery dataset instead of the validation dataset; the discovery dataset is less affected by unwanted variation.

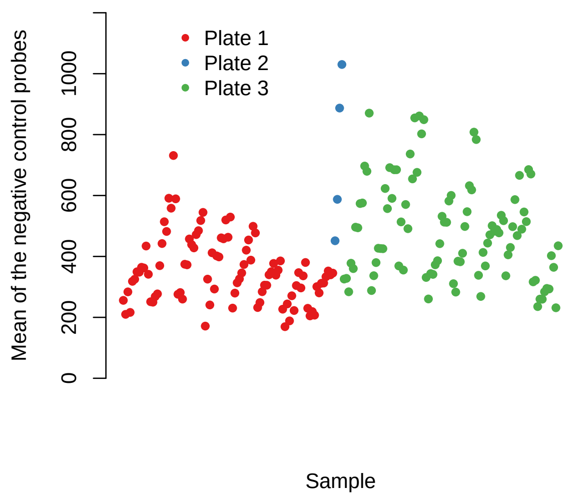

(a)

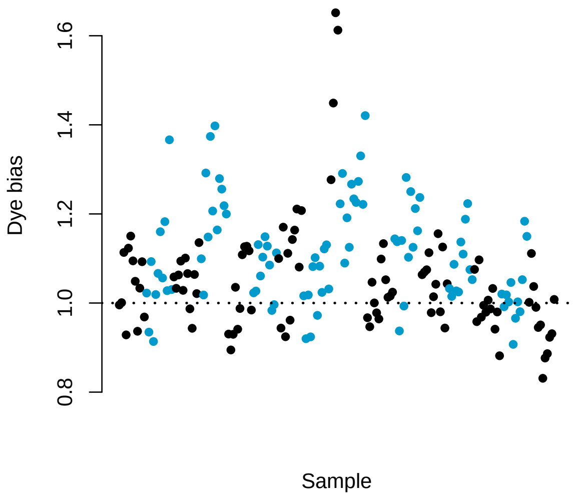

(b)

Supplementary Figure S5. Plate effects and dye bias for the AML dataset (a) The means of the negative control probes is correlated with the processing plate (96 samples) indicating that background intensity is affected by batch. (b) We measure dye bias by taking the ratio of the negative control probes in the green channel and the negative control probes in the red channel. A value of 1 means that there is no dye-bias. We plot the dye bias (y-axis) for samples ordered by plate and then by slide (x-axis). The plate order is the same as (a). We use two different alternating colors to differentiate the slides. We observe that dye bias is orthogonal to plate effect and highly slide dependent.

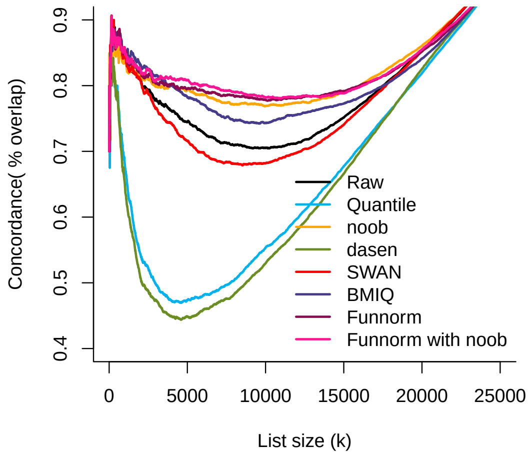

(a)

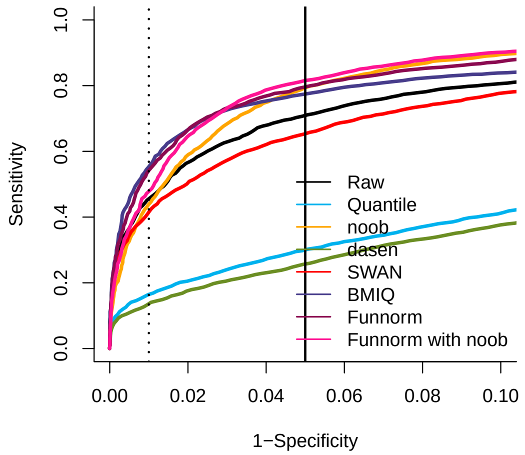

(b)

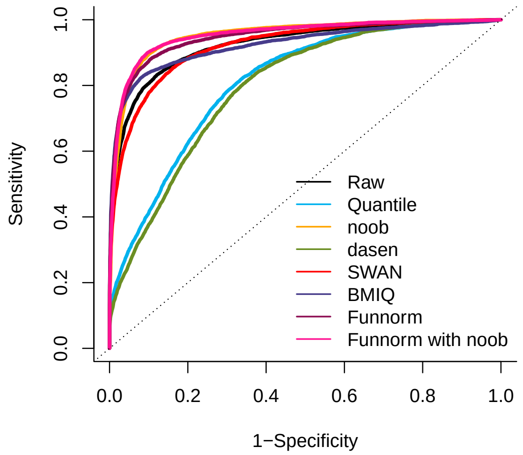

(c)

Supplementary Figure S6. Improvements in replication of tumor subtype heterogeneity. (a) Like Figure 4a. (b) Like Figure 4b but for all normalization methods. (c) Like (b) but for the full range of specificity

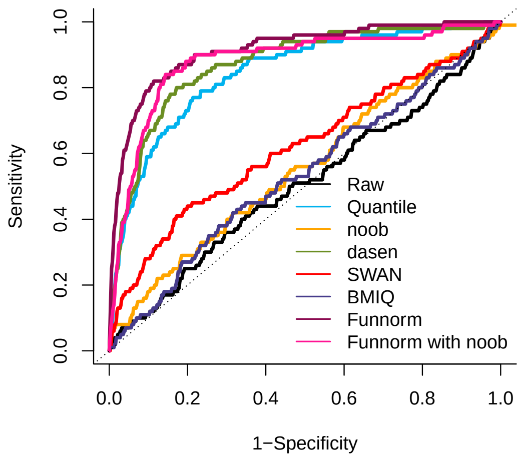

(a) Ontario-Blood

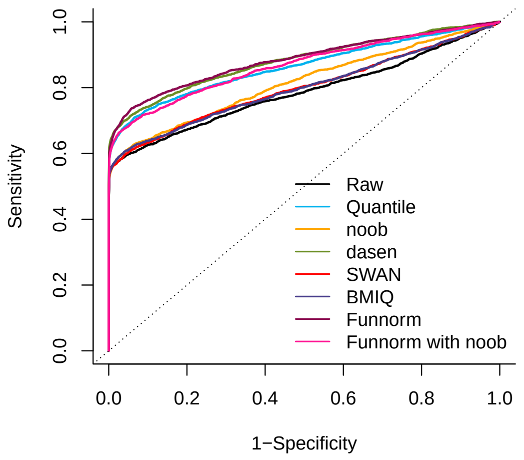

(b) Ontario-Gender

Supplementary Figure S7. Improvements in blood samples, all methods Like Figure 5, but for all normalization methods we assess.

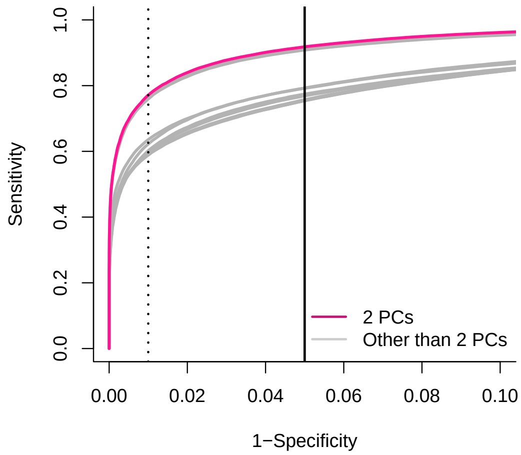

(a) Ontario-EBV

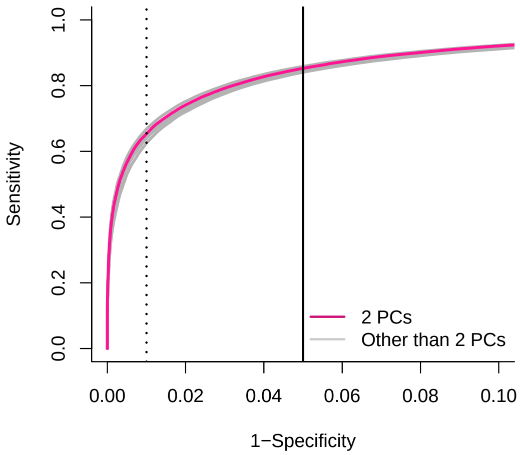

(b) TCGA KIRC

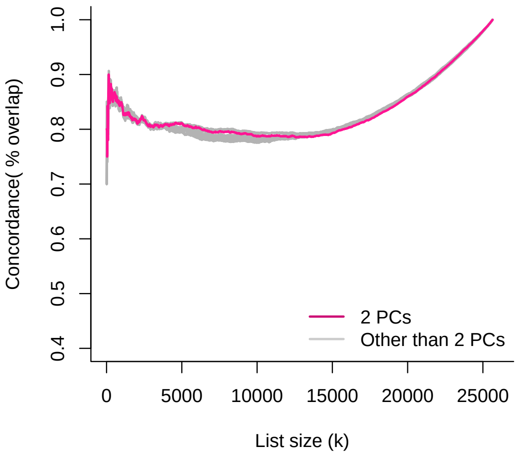

(c) TCGA AML

Supplementary Figure S8. The impact of the number of principal components. (a) Like Figure 2a. (b) Like Figure 3a. (c) Like Figure 4a, but showing the difference between using $m = 2$ components and other choices of $m \leq 10$.

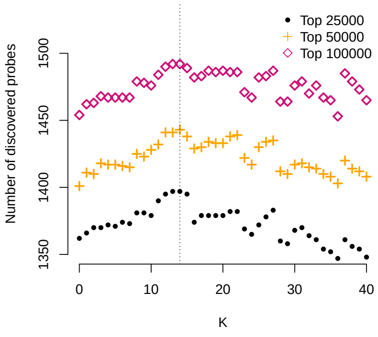

(a) Ontario-EBV Discovery

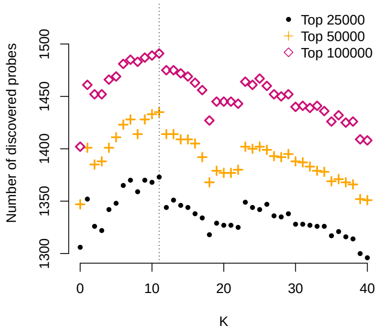

(b) Ontario-EBV Validation

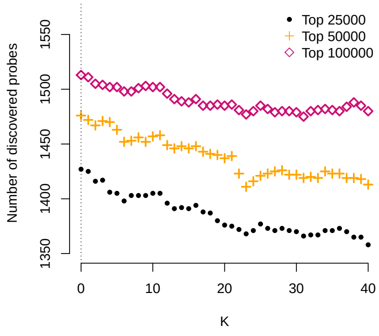

(c) TCGA KIRC Discovery

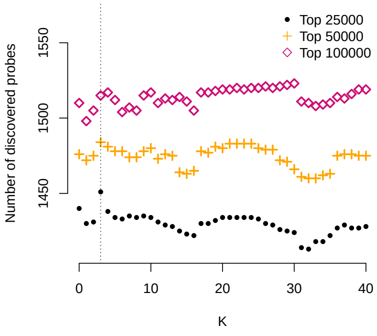

(d) TCGA KIRC Validation

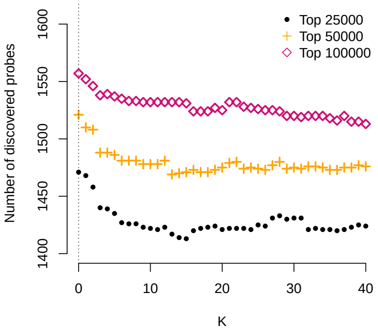

(e) TCGA AML

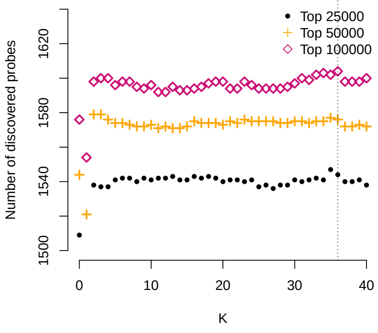

(f) Ontario-Blood Discovery

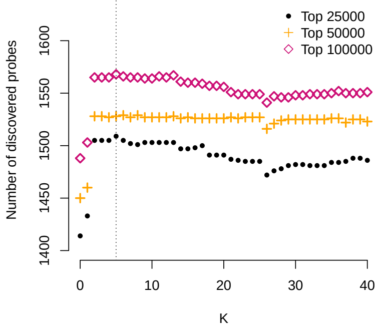

(g) Ontario-Blood Validation

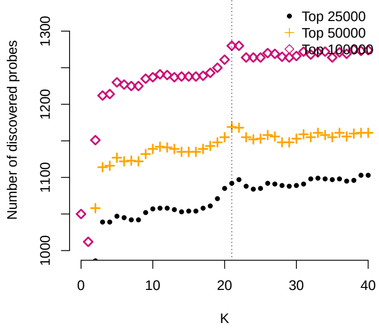

(h) Ontario-Gender

Supplementary Figure S9. RUV tuning plots. Selection of the tuning parameter for RUV.

Supplementary Material

In the following text we describe exhaustively the transformations that we applied to the control probes to create the control probe summaries that were used throughout the paper as covariates in the functional normalization. Note that we chose the transformations by considering the recommendations made in the GenomeStudio Methylation manual [Ill, 2010].

- For "Bisulfite Conversion I" probes, 3 probes (C1,C2,C3) are expected to have high signal in the green channel in case the bisulfite conversion reaction was successful, and similarly 3 additional probes (C4,C5,C6) are expected to have high signal in the red channel. We therefore consider these 6 intensities and take the mean as a single summary value.
- For "Bisulfite Conversion II" probes, 4 probes are expected to have high intensities in the red channel in case the bisulfite conversion reaction was successful. Therefore we consider the mean of these 4 intensities as a single summary value.
- For the "Extension" control probes, 2 probes must be monitored in the red channel (A,T) and 2 probes must be monitored in the green channel (C,G). We consider the 4 raw intensities as output values for a total of 4 summary values.
- For the "Hybridization" probes, the 3 probes have to be monitored in the green channel. We consider the raw intensities as output values, corresponding to low, medium and high hybridization signals, for a total of 3 summary values.
- For the "Staining" probes, we select the green intensity of the probe that is expected to have high intensity in the green channel, and similarly for the probe that is expected to have high intensity in the red channel. This results in 2 summary values.
- For "Non-polymorphic" controls, we consider the 2 probes that are expected to be high in the green channel (C and G) and the two probes that are expected to be high in the red channel (A and T). We consider the 4 raw intensities as output for a total of 4 summary values.
- "Target removal" probes have to be monitored only in the green channel. We use the raw intensities for the 2 probes as output values for a total of 2 summary values.
- For "Specificity II" probes, we monitor the 3 probes in both the green and red channels, for a total of 6 output values. The green channel is expected to be low and the red channel to be high, so we also form the ration of the mean of the green channel to the mean of the red channel as output value. This results in 7 summary values.
- For "Specificity I" probes, 3 probes are expected to have high signal in the green channel, and 3 different probes are expected to have high signal in the red channel. For each trio, we take the raw intensities in the corresponding "good" channel, giving us 6 output values. For each trio, we also consider the signal-to-noise ratio

by taking the ratio of the mean of the 3 intensities measured in the good channel (high signal expected) and the mean of the three intensities in the opposite channel (low signal expected). This gives us 2 ratio summaries. We also consider the mean of these two ratios, giving us 1 additional output value. This results in a total of 9 summary values.

- "Normalization" probes: probes targeting A bases (32) and T bases (61) have to be monitored in the red channel and probes targeting G (32) and C (61) have to be monitored in the green channel. For each type (A,C,T,G), we consider the mean of the intensities in their corresponding channel, that we denote by $normA$, $normC$, $normT$ and $normG$ (4 output values). Moreover, we consider the ratio $(normC + normG)/(normA + normT)$ as a surrogate for dye bias computed with positive controls (1 additional output value), for a total of 5 summary values.
- For the Out-of-band probes (Oob), we first take the 1st, 50th and 99th percentiles of the 92,596 green intensities (3 output values). Because the variation seen in the green Oob probes is similar to the that of the red Oob probes, we omit the latter. Nevertheless, we consider the ratio of the median of the 92,596 green intensities and the median of the 178,406 red intensities, as a surrogate for dye bias. This results in a total of 4 summary values.

References

GenomeStudio Methylation Module v1.8 User Guide. Illumina, 2010.
